# Novel strategy for multi-material 3D bioprinting of human stem cell based corneal stroma with heterogenous design

**DOI:** 10.1016/j.mtbio.2023.100924

**Published:** 2023-12-22

**Authors:** Paula Puistola, Susanna Miettinen, Heli Skottman, Anni Mörö

**Affiliations:** a Eye Regeneration Group, Faculty of Medicine and Health Technology, Tampere University, Tampere 33520, Finland; b Adult Stem Cell Group, Faculty of Medicine and Health Technology, Tampere University, Tampere 33520, Finland; c Research, Development and Innovation Centre, Tampere University Hospital, 33520 Tampere, Finland

**Keywords:** Multi-material bioprinting, 3D bioprinting, Heterogenous design, Cornea, Human stem cells

## Abstract

Three-dimensional (3D) bioprinting offers an automated, customizable solution to manufacture highly detailed 3D tissue constructs and holds great promise for regenerative medicine to solve the severe global shortage of donor tissues and organs. However, uni-material 3D bioprinting is not sufficient for manufacturing heterogenous 3D constructs with native-like microstructures and thus, innovative multi-material solutions are required. Here, we developed a novel multi-material 3D bioprinting strategy for bioprinting human corneal stroma. The human cornea is the transparent outer layer of your eye, and vision loss due to corneal blindness has serious effects on the quality of life of individuals. One of the main reasons for corneal blindness is the damage in the detailed organization of the corneal stroma where collagen fibrils are arranged in layers perpendicular to each other and the corneal stromal cells grow along the fibrils. Donor corneas for treating corneal blindness are scarce, and the current tissue engineering (TE) technologies cannot produce artificial corneas with the complex microstructure of native corneal stroma. To address this, we developed a novel multi-material 3D bioprinting strategy to mimic detailed organization of corneal stroma. These multi-material 3D structures with heterogenous design were bioprinted by using human adipose tissue -derived stem cells (hASCs) and hyaluronic acid (HA) -based bioinks with varying stiffnesses. In our novel design of 3D models, acellular stiffer HA-bioink and cell-laden softer HA-bioink were printed in alternating filaments, and the filaments were printed perpendicularly in alternating layers. The multi-material bioprinting strategy was applied for the first time in corneal stroma 3D bioprinting to mimic the native microstructure. As a result, the soft bioink promoted cellular growth and tissue formation of hASCs in the multi-material 3D bioprinted composites, whereas the stiff bioink provided mechanical support as well as guidance of cellular organization upon culture. Interestingly, cellular growth and tissue formation altered the mechanical properties of the bioprinted composite constructs significantly. Importantly, the bioprinted composite structures showed good integration to the host tissue in *ex vivo* cornea organ culture model. As a conclusion, the developed multi-material bioprinting strategy provides great potential as a biofabrication solution for manufacturing organized, heterogenous microstructures of native tissues. To the best of our knowledge, this multi-material bioprinting strategy has never been applied in corneal bioprinting. Therefore, our work advances the technological achievements in additive manufacturing and brings the field of corneal TE to a new level.

## Highlights

• The novel strategy for multi-material bioprinting was inspired by the native human corneal stroma.
• Cell-laden and acellular bioinks with different stiffnesses were printed in alternating filaments.
• The cell-laden bioink allowed cellular growth and tissue formation within filaments.
• The acellular bioink guided cellular growth and enhanced structural stability.
• The 3D structures with heterogenous design mimicked the organization of corneal stroma.

## Graphical abstract

**Figure:**
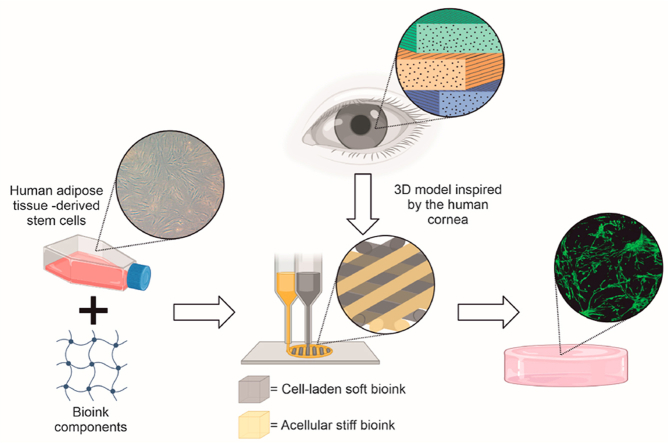

## 1 Introduction

The human body is a highly complex system composed of heterogenous tissues and organs. When the body encounters its limits in regeneration capabilities, donor tissues and organs are needed. One of the most essential senses for humans is vision. Clear vision is provided by the human cornea which is the outermost layer of the eye [1]. If the cornea is damaged, it can lead to corneal blindness. Losing vision has serious effects on the quality of life of individuals, affecting their independence, employment, mental health and social function [2]. The human cornea is one of the most transplanted tissues, and yet there is a severe shortage of donor corneas for treating corneal blindness. In fact, only 1 patient out of 70 in need receives the donor cornea, creating a serious need for artificial corneas [3]. The corneal stroma comprises 90 % of the human cornea and has the key role in transparency and mechanical strength providing clear vision. These crucial features are based on the highly organized collagen type I fibrils which are arranged in layers perpendicular to each other. Human corneal stromal keratocytes (hCSKs) grow between the collagen fibrils, maintaining the corneal stroma and its properties [1]. Thus, it is essential to mimic this complex and precise microstructure to fabricate artificial corneas to benefit the patients in need.

Three-dimensional (3D) bioprinting has become the state-of-the-art biofabrication technology to manufacture artificial tissues with the cellular architecture and spatial organization mimicking the native tissues. In 3D bioprinting, a bioink composing of cells and biomaterials is printed layer-by-layer based on a pre-designed 3D model in automated and repeatable manner [4]. Therefore, 3D bioprinting technology offers a highly potential solution to the severe shortage of donor corneas. Several 3D bioprinting technologies have been explored to fabricate the human cornea, including extrusion-based [5,6], inkjet-based [7], laser-assisted [8] and stereolithographic bioprinting [9,10]. Prior research in corneal 3D bioprinting shows that several technical aspects of fabricating artificial corneas are well-established. However, many previous studies focus on the curved shape of the cornea [5,7,11,12], or its mechanical and optical properties [10], and fail to show efficient tissue formation after printing. Importantly, the field lacks research in mimicking the heterogenous microstructure of the corneal stroma, including the detailed organization of the collagen fibrils essential for clear vision. Therefore, novel strategies are needed to combine the mechanical stability with hierarchical composition of the native corneal stroma in corneal tissue engineering (TE).

Even though 3D bioprinting has tremendous potential for regenerative medicine, novel multi-material solutions combining multiple biomaterials into one structure is required to mimic the highly heterogenous composition of native tissues [13,14]. Widely accepted method to fabricate heterogenous 3D constructs is to combine thermoplastic polymer, such as polycaprolactone (PCL), with a hydrogel bioink [[15], [16], [17]]. As an emerging technology, melt-electrowetting (MEW) has been explored to fabricate thermoplastic polymer framework with high organization to mimic the corneal stroma [18], and it can also be combined with hydrogel-based bioinks in bioprinting [19]. Thermoplastic polymer frameworks provide mechanical support and can guide cellular growth, making it a potential strategy to mimic the organization of the corneal stroma. However, the use of thermoplastic polymers in cornea TE is limited by the requirements for the transparency [20]. Moreover, they often require surface modification to demonstrate sufficient cell attachment [21]. To fabricate heterogenous structures without thermoplastic polymers, it is possible to print different bioinks or cell types in different layers of the 3D construct [22,23]. Bioprinting alternating layers of bioinks with different compositions has been previously explored for cornea using laser-assisted bioprinting [8]. However, the precise organization of corneal stromal fibrils and cellular organization within layers cannot be achieved with this approach. More recent approaches to achieve cellular organization include encapsulating mechanically fragmented electrospun microfibers within gelatin-methacrylamide (GelMA) [24] and patterning cells ultrasonically in alginate or GelMA [25]. Nevertheless, these methods have not been applied to corneal TE. In addition, even though these methods support cell alignment, the drawbacks include a decrease in the transparency [24], and the lack of sufficient tissue formation [24,25]. Moreover, a question remains in the mechanical stability and handling of the structures which enables transplantation [25].

Here, we developed a novel strategy for multi-material 3D bioprinting to manufacture native-like tissue constructs with heterogenous design that can withstand handling. To the best of our knowledge, multi-material approach has never been applied in corneal stroma bioprinting. The developed strategy was applied to bioprinting of a cell-laden and acellular hyaluronic acid (HA) -based bioink and human adipose tissue -derived stem cells (hASCs). The bioinks had different crosslinking densities, and thus, different stiffnesses. The heterogenous design of the 3D structures was inspired by the native corneal stromal microstructure. Therefore, to mimic the organization of collagen fibrils and corneal stromal cells, the cell-laden and acellular bioink with different stiffnesses were printed as alternating filaments in perpendicular layers to create composite structures. Bioinks were characterized, and the mechanical properties, transparency, handling and swelling of the bioprinted structures were investigated. Cell orientation and growth as well as tissue formation within the bioprinted composite structures were studied to evaluate the ability of the multi-material bioprinting strategy to mimic the heterogenous microstructure of native corneal stroma. Finally, the feasibility of the composite structure for corneal TE in an *ex vivo* porcine corneal organ culture model was demonstrated.

## 2 Materials and methods

### 2.1 Synthesis of the crosslinking components

HA-Aldehyde (HA-ALD) was synthesized according to the previously reported protocol [26]. The synthesis of HA with dopamine and carbohydrazide modification (HA-DA-CDH) was conducted as reported previously [27]. The synthesized and lyophilized components were stored at −20 °C before use.

### 2.2 Preparation of the bioinks

The bioinks were mixed as previously reported [28] with slight modifications. Briefly, the crosslinking components HA-DA-CDH and HA-ALD were diluted in 1*X* PBS (Dulbecco's Phosphate Buffered Saline, DPBS, Carl Roth). For the soft bioink, both components were dissolved into concentration of 9 mg/ml, whereas for the stiff bioink concentration of 14 mg/ml was used. For printing hASCs, the cells were resuspended in the desired volume of cell culture medium and mixed into the soft bioink.

### 2.3 3D bioprinting setup

After mixing, the bioinks were transferred in 30 cc barrels (Nordson EFD) with pistons and 32 G blunt needles (0.5″, CELLINK). Before printing, the soft and stiff bioinks were pre-crosslinked for 70 min and 40 min, respectively. Pre-crosslinking times were optimized for bioinks based on the difference in crosslinking component concentrations and crosslinking rates to obtain optimal biofabrication window. Printing was done with extrusion-based bioprinter 3D-Bioplotter (EnvisionTEC) on 35 mm petri dishes (TC-treated, Corning®) at room temperature (20 °C). The 3D models with 80 μm slicing interval were created with Perfactory RP software. The inner structures of the printed layers were designed in Visual Machines software. The printing pressure and speed for soft and stiff bioinks were 1.0 bar and 4.0 bar, and 7 mm/s and 6 mm/s, respectively. The pre-flow values of the soft and stiff bioinks were set to 0.1 s and 0.3 s, and the post-flow values to 0.1 s and 0.2 s, respectively. The biofabrication window of 1 h was obtained for both bioinks. The printed structures were stabilized at 37 °C with 5 % CO_2_ at humid environment before adding cell culture medium or 1*X* PBS to cover the structure.

### 2.4 Bioink characterization

To analyze the printability and shape fidelity of the bioinks, grids with six perpendicular layers and 2.5 mm distance between filaments were printed and analyzed immediately after printing and after 7 days as described previously by Mörö et al. [28]. The thickness of the filaments and the pore factors of the grids were determined from the images by using ImageJ Fiji software. Nine filaments as well as six pores from four grid replicates were analyzed in both timepoints (n = 4).

Shear-thinning properties of the bioinks, including cell-laden soft bioink, were analyzed by measuring their viscosities with HR-2 Discovery hybrid rheometer (TA Instruments). Continuous flow rate with shear rate ranging from 0.01 to 10 s^−1^ and 20 mm parallel plate geometry with 1 mm gap were used. The bioinks were prepared as described above. The measurements were carried out within 1 h after starting the first measurement, 500 μl bioink sample size was used, and three replicates per bioink were measured (n = 3).

The transparency of the bioinks was analyzed by measuring their transmittance with a UV/VIS spectrophotometer (Lamda 35 UV/VIS Spectrophotometer, Perkin Elmer). The bioinks were prepared as described above. Before 1 h pre-crosslinking, the bioinks were transferred into cuvettes and centrifuged at 1000 g for 1 min to remove excess air. Air was used as blank in the transmittance measurements.

Sufficient adhesion between two bioinks is crucial for the handling and structural stability of the bioprinted construct. In a simple gel block fusion test, the examined hydrogel disc is cut in half using a scalpel, followed by rejoining the pieces back together and observing the healing process visually [29]. The bioinks were prepared as described above, and red food dye was mixed to the stiff bioink to a concentration of 4.2 μl/ml. Soft and stiff bioinks were pre-crosslinked in syringes for 24 h. Thereafter, the gel discs were pushed out and cut in half, and the halves from different bioinks were combined. After 24 h of self-healing at room temperature, the adhesion between the halves was studied visually and by lifting the structures with a spatula and pulling the halves apart with tweezers. The adhesion was further studied by conducting compression tests with HR-2 Discovery hybrid rheometer as described by Mörö et al. [28]. The halves from different bioinks (soft + stiff) were combined and after 24 h, axial compression with 12 mm parallel plate geometry and displacement rate of 1 mm min^−1^ was carried out. Same measurements were conducted to soft + stiff disk immediately after joining the halves, as well as uncut disks from soft and stiff bioinks. All measurements were carried out in triplicates (n = 3). The force curves were plotted against compression percentage.

### 2.5 Cell culture

Due to the abundancy and immunomodulatory properties [30], differentiation capability towards hCSKs [[31], [32], [33], [34], [35], [36]], potential in preserving the corneal transparency [37] and promising results from human clinical studies for treating corneal stroma pathologies [38,39], hASCs were selected as a cell source in this study. The hASCs were isolated from subcutaneous adipose tissue samples. The isolation, expansion and characterization of the hASCs has been previously described by Refs. [8,40]. Thereafter, hASCs were cultured in basic medium (BM) composed of DMEM/F-12 supplemented with 5 % human serum (Serana), 1 % penicillin/streptomycin (Gibco™) and 1 % Glutamax (Thermo Scientific™) at 37 °C with 5 % CO_2_. The hASCs were cultured and passaged in T75-flasks (Nunc™ EasYFlask™, Thermo Scientific) until used for printing at passage 5 with a cell density of 9×10^5^ cells ml^−1^ in the soft bioink. The printed cell-laden structures were cultured in BM.

### 2.6 Printing of the 3D structures

**Fig. 1:**
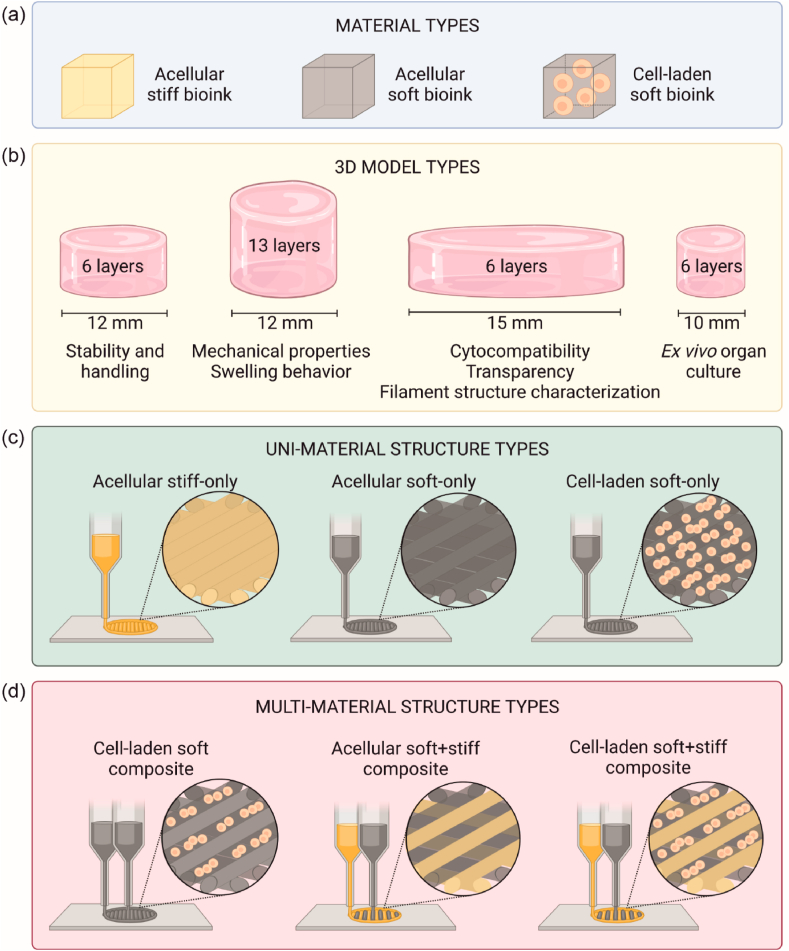
Schematic illustration of different bioinks and 3D bioprinted structure types. (a) Different material types used for 3D bioprinting (b) Dimensions of the 3D model types used in analyses. (c) Uni-material structure types consisted of either acellular stiff or soft bioink, or cell-laden soft bioink. (d) Soft and stiff bioinks were bioprinted as multi-material composites. The composites were either completely acellular or cell-laden with the acellular filament printed from soft or stiff bioink.

The printing setup used for bioprinting of 3D structures is described in section 2.3. The different bioinks used for bioprinting are summarized in Fig. 1(a). Four cylindrical 3D models were designed for different analyses (Fig. 1(b)). The 3D models were bioprinted with the bioinks into uni-material structures (Fig. 1(c)) and multi-material structures that are from here on referred to as composites (Fig. 1(d)). For stability and handling tests, acellular soft-only uni-material structures and acellular soft + stiff composites were printed. For analyzing mechanical properties and swelling behavior, acellular soft-only and stiff-only uni-material structures as well as acellular and cell-laden soft + stiff composites were printed. For exploring the visual transparency, cell-laden soft composites and soft + stiff composites were printed. For transmittance measurements, acellular and cell-laden soft + stiff composites were printed. For determining cytocompatibility, cell-laden soft composites and soft + stiff composites were printed. For characterizing filament structure, cell-laden soft + stiff composites were printed. In *ex vivo* organ culture, cell-laden soft + stiff composites were used.

For all printed structures, the inner pattern of one printed layer was a continuous filament, and the filaments were printed perpendicularly in alternating layers. The filament strand distance was 0.35 mm in uni-material structures and 0.7 mm for alternating filaments in composites. Two contours with 0.2 mm distance were printed in each 3D structure. In composites, the contours were printed from the acellular stiff or soft bioink. The stabilization time was 90 min for all 3D structure types.

### 2.7 Analysis of the 3D structures

The stability and handling of the bioprinted structures in 1*X* PBS was analyzed after 7 days post-printing. For the analysis, the PBS was removed, and the structures were handled with a spatula. The stability of the printed structures was evaluated visually.

Visual transparency of the structures was analyzed after 1 and 7 days post-printing. The structures were cultured in BM and rinsed with 1*X* PBS before taking photographs against a paper with text in natural lighting. Transparency was further studied with absorbance measurements of acellular and cell-laden composites after 1 and 7 days post-printing. After culturing the structures in BM, 5 mm cylindrical pieces were punched, rinsed with 1*X* PBS and measured on 96 well plate with Spark® multimode plate reader (Tecan) at wavelength range of 400–700 nm. Measurements were carried out in triplicates (n = 3). The transmittance values were calculated from absorbance values as described previously by Kim et al. [41] and Kutlehria et al. [10]. PBS was used as control.

The mechanical properties of four structure types cultured in BM were assessed with HR-2 Discovery hybrid rheometer by measuring the amplitude and frequency sweeps on day 1, 7 and 14 after printing. 12 mm parallel plate geometry with a gap of 1 mm was used. Amplitude sweeps were performed with constant frequency of 1 Hz and the oscillation strain ranging from 0.01 % to 50 %. Frequency sweeps were performed with frequency ranging from 0.1 Hz to 10 Hz and with a constant strain of 1 % based on the amplitude sweeps. Amplitude sweeps were performed once per structure type per timepoint, and frequency sweeps were performed in triplicates (n = 3). The printed structures were trimmed before measuring to prevent over- or underfilling.

Swelling behavior of four structure types was analyzed by weighing the structures on petri dishes on day 1, 7 and 14. The initial weight was determined by measuring the weight of the printed structure after 1 h stabilization period and 30 min immersion period in BM. All the measurements were done in triplicates (n = 3).

To evaluate the post-printing stability of the soft and stiff filaments within the composites, soft + stiff composites with 0.5 μm fluorescent particles (yellow-green, FluoSpheres™, Invitrogen) were printed. Fluorescent particles were mixed in the soft bioink as 0.17 % (v/v). The printed structures in 1*X* PBS were imaged after 1 and 7 days post-printing with Leica Dmi8 (Leica Microsystems). From the z stack images, three layers were separated and converted as maximum intensity projections (MIPs) to illustrate the formation and stability of the soft filaments within the composites. The image editing was done in ImageJ Fiji.

### 2.8 Cell viability and proliferation evaluation

Cell viability and proliferation in the printed structures was evaluated with LIVE/DEAD® Viability/Cytotoxicity Kit for mammalian cells (Thermo Fischer Scientific) and PrestoBlue™ viability assay (Thermo Fischer Scientific). These analyses were performed on two cell-laden composites. LIVE/DEAD staining was performed on day 1 and 7 after printing, and PrestoBlue™ assay on day 1, 7 and 14. LIVE/DEAD staining was carried out according to the instructions from the manufacturer [42]. After 30 min incubation at 37 °C, the samples were imaged with confocal microscope (LSM 800, Zeiss). PrestoBlue™ staining was carried out according to the manufacturer's instructions [43]. After 1 h incubation at 37C°, the absorbance of three replicates (n = 3) was measured with a multiplate reader (Victor2 Microplate reader, Perkin Elmer).

### 2.9 Immunofluorescence staining

To analyze the cell morphology and maturation as well as tissue formation after printing, immunofluorescence (IF) staining with phalloidin-tetramethylrhodamine B isothiocyanate (Sigma-Aldrich) and anti-connexin 43 (rabbit, Sigma-Aldrich) were used. Phalloidin stains the actin of the cytoskeleton and connexin 43 stains the gap junctions between cells. In addition, Hoechst 33,342 (Invitrogen) was used to stain the cell nuclei. The cell morphology and tissue formation were analyzed from the two cell-laden composites. Printed samples were fixed on day 1, 7 and 14 after printing with 4 % paraformaldehyde for 1 h at room temperature, followed by washing again with 1*X* PBS three times. Thereafter, the fixed samples were immersed in 5 % bovine serum albumin (BSA, Sigma) in 1*X* PBS with 0.1 % Triton X-100 (Sigma) over night at room temperature for permeabilization and blocking. Primary antibody solution with anti-connexin 43 1:100 was prepared in 5 % BSA in 1*X* PBS, and the samples were incubated at 4 °C for 3 days. Next, the samples were washed with 1*X* PBS for 2 days. Secondary antibody solution containing anti-rabbit Alexa 488 (Molecular Probes) 1:400, phalloidin 1:100 and Hoechst 1:1200 was prepared in 5 % BSA in 1*X* PBS, and the samples were incubated at room temperature overnight. Finally, the samples were washed again with 1*X* PBS for 1 day and mounted with Vectashield® Antifade Mounting medium (Vector Laboratories) on glass bottom dishes (MatTek). The samples were imaged with Zeiss confocal microscope and the z stack images were deconvoluted with Huygens Essential software (Scientific Volume Imaging) and edited in ImageJ Fiji as MIPs. 3D views of the stack images were visualized in Imaris (Oxford Instruments).

### 2.10 Porcine cornea *ex vivo* organ culture and analysis

The porcine cornea *ex vivo* organ culture model was used as a transplantation platform as previously described with slight modifications [8,27]. After porcine corneas were dissected from the eyes in aseptic conditions and disinfected, they were cultured in BM supplemented with 0.25 μg/ml amphotericin B (Thermo Fisher Scientific) at 37 °C with 5 % CO_2_ overnight. On the next day, cell-laden soft + stiff composites with yellow-green fluorescent particles as 0.17 % (v/v) in the stiff bioink and hASCs in soft bioink were printed. The structures were allowed to stabilize 4 h at 37 °C with 5 % CO_2_ before transplantation into dissected porcine corneas. Transplantation was carried out on a Barron artificial anterior chamber (Katena products Inc., USA). A crescent knife (Bauch&Lomb Inc. USA) was used to make a stromal pocket to the center of the cornea and a 5 mm piece of the composite was punched and transplanted into the pocket. The printed structures were further stabilized in the pockets for 2 h before immersing corneas in BM. The *ex vivo* organ culture was carried out for 14 days (n = 5). Porcine cornea without stromal pocket was used as control (n = 1).

For analysis, the corneas were fixed with 4 % PFA solution for 3 h at RT, washed with 1*X* PBS for 2 h and immersed in 20 % sucrose solution overnight at 4 °C. On the next day, the corneas were transferred from sucrose to Tissue-Tek OCT (Science Services, Germany) and incubated overnight at 4 °C. Thereafter, the corneas were snap frozen at liquid nitrogen and stored at −80 °C. Cryosections of 10 μm were prepared with a microtome on Epredia™ Superfrost™ Plus slides (Epredia). The cryosections were air-dried for 30 min before IF or hematoxylin and eosin (H&E) staining.

Mouse anti-human STEM121 (Takara) 1:80 primary antibody was used in IF staining to detect printed hASCs in porcine cornea *ex vivo* organ cultures after transplantation. Secondary antibody solution contained anti-mouse Alexa 647 (Molecular Probes) 1:400, phalloidin 1:100 and Hoechst 1:1000. The cryosections were mounted with Prolong™ Gold Antifade Mountant (Invitrogen) and imaged with Leica Dmi8. H&E staining was done with KD-RS3 automatic slide stainer (KEDEE) with a standard protocol for cryosections, with Mayers hematoxylin Plus (Histolab) and 0.2 % eosin (Histolab) incubations of 3 min and 30 s, respectively. After staining, the cryosections were mounted with DPX new mounting medium (Sigma-Aldrich) and DAKO coverslipper (Agilent) and imaged with Nikon Eclipse TE200S microscope (Nikon Instruments).

### 2.11 Statistical analysis

The statistical significance of shape fidelity analysis, frequency sweeps and PrestoBlue™ cell proliferation analysis were determined by Mann-Whitney *U* test. *P*-values ≤0.05 were considered statistically significant. SPSS software (IBM) was used for the statistical data analysis. All data is presented as mean values with ±standard deviation.

### 2.12 Ethical issues

This study was conducted under approvals from Regional Ethics Committee of the Expert Responsibility area of Tampere University Hospital that allow to extract and use hASCs for research purposes (R15161).

## 3 Results

### 3.1 Both bioinks demonstrate excellent printability and shape fidelity

**Fig. 2:**
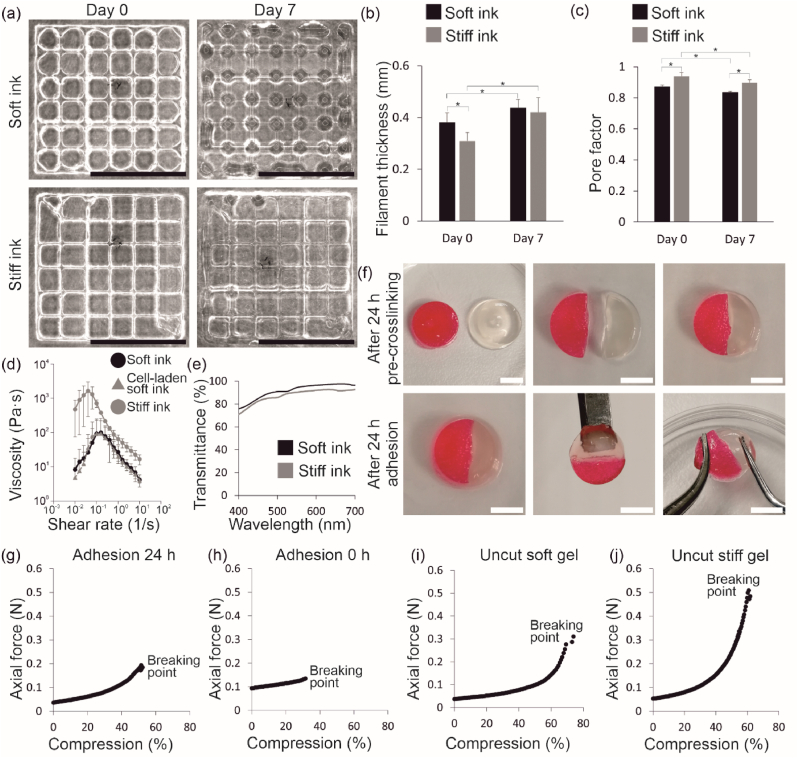
Both bioinks demonstrated excellent printability and shape fidelity. (a). Images of the printed filament structure of the soft and stiff bioinks on day 0 and 7. Shape fidelity was assessed from (b) filament thickness and (c) pore factor (*p ≤ 0.05). (d) Shear-thinning behavior of the soft and stiff bioink, and cell-laden soft bioink. (e) Transmittance of the soft and stiff bioinks. Adhesion between the bioinks was evaluated after 24 h with (f) gel block fusion test and compression test where (g) adhesion after 24 h was compared to (h) adhesion after 0 h. (i) Uncut soft gel and (j) uncut stiff gel were used as controls in compression test. Scalebars (a) 10 mm and (f) 5 mm.

The printability was assessed from grid structures. The grids were clear, and the filaments were uniform with both bioinks (Fig. 2(a)). The used printing pressure of the stiff bioink was 4-times higher than the pressure of the soft bioink. After 7 days in PBS, the grids were still visible and intact, indicating great shape fidelity. Shape fidelity was further assessed by analyzing filament thickness (Fig. 2(b)) and pore structure of the grids (Fig. 2(c)). The filament thicknesses on day 0 and 7 were 0.38 ± 0.04 mm and 0.44 ± 0.03 mm for the soft ink, respectively, and 0.31 ± 0.03 mm and 0.42 ± 0.06 mm for the stiff ink, respectively. The filament thickness of the stiff bioink on day 0 was significantly lower compared to the soft bioink (p ≤ 0.05), which indicates better shape fidelity during printing. The filament thickness of both bioinks increased during the 7 days due to swelling and there was no significant difference between the filament thicknesses of the bioinks on day 7 (p ≤ 0.05). The Pr demonstrates the shape of the pore, with Pr = 1 indicating perfect rectangular shape and Pr < 1 indicating more circular shape. The Pr of the soft bioink was significantly lower (p ≤ 0.05) compared to the Pr of the stiff bioink on both day 0 and 7 (Fig. 2(c)). This indicates more circular shape of pores for the soft bioink, which is supported by the images of the grid structures (Fig. 2(a)). On day 0, the Pr values of the soft and stiff bioinks were 0.87 ± 0.01 and 0.94 ± 0.02, respectively. On day 7, the Pr values of the bioinks decreased to 0.84 ± 0.01 and 0.90 ± 0.02, respectively. Even though the decrease in the Pr values demonstrate the pores becoming more circular due to filament swelling, the Pr values remained close to 1.

Both bioinks demonstrated shear-thinning properties as the viscosity decreased when the shear rate was increased (Fig. 2(d)). The peak viscosities of the soft and stiff bioinks were 99 ± 152 Pa s and 1628 ± 1339 Pa s, which indicates that the material is softer and easier to extrude. Thus, the soft bioink demonstrated more suitable rheological properties for cell printing since high printing pressure was not required to initiate extrusion of the material. To compare, the viscosity of the stiff bioink was over 10-times higher than the one of the soft bioink. Nevertheless, the viscosity of the stiff bioink decreased when the shear rate was increased, and therefore the excellent printability shown in Fig. 2(a) was supported by the viscosity measurements. However, higher viscosity of the stiff bioink indicates high shear stress when printing, which is supported by the printing pressure data. Therefore, the stiff bioink was not chosen for cell printing with high resolution printing needle. Importantly, the addition of cells to the soft bioink did not hamper its shear-thinning properties (Fig. 2(d)) nor significantly alter the peak viscosity where it was 92 ± 125 Pa s for the cell-laden soft bioink.

The transparency of the bioinks was analyzed by measuring their transmittance (Fig. 2(e)). The transmittance of the soft and stiff bioinks was 75–97 % and 70–92 %, respectively, within the visible light wavelength range. The higher crosslinking density of the stiff bioink decreased the transmittance value slightly compared to the soft bioink but the difference was not significant and both bioinks demonstrated excellent transparency. The adhesion between the two bioinks was studied with a gel block fusion test by combining gel disk halves from soft and stiff bioink together (Fig. 2(f)). After 24 h, the disk halves were firmly attached to each other. The adhesion between them was strong enough to hold the structure's own weight when lifted with a spatula and to prevent rupturing when the structure was pulled apart with tweezers. Furthermore, when further evaluating the adhesion between the soft and stiff bioinks with a compression test, the gels with 0 h adhesion were notably weaker compared to gels with 24 h adhesion (Fig. 2(g and h)). After 24 h adhesion, the axial force and compression percentage at the breaking point were 0.19 ± 0.06 N and 51.4 ± 3.6 %, respectively. After 0 h adhesion, the axial force and compression percentage at the breaking point were 0.13 ± 0.003 N and 31.5 ± 1.9 %, respectively. Thus, the gels withstood higher axial force as well as deformation after 24 h adhesion. This data together with the gel block fusion test demonstrates sufficient adhesion between the different bioinks, and thus, bioink filaments of the printed 3D composite. Due to this, the filaments printed from different bioinks do not pull apart and the multi-material composite remains solid. Moreover, the compression test of uncut soft and stiff control gels (Fig. 2(i and j)) demonstrated that both gels withstand similar compression (69.3 ± 3.9 % for soft, 60.8 ± 3.2 % for stiff), even though the stiff gel withstands 1.7-times higher axial force (0.48 ± 0.15 N) than the soft gel (0.28 ± 0.13 N). Therefore, the crosslinking density and bioink stiffness does not hamper the deformation capability.

### 3.2 Multi-material 3D bioprinting strategy and cellular growth enhances the handling and mechanical stability of the 3D bioprinted constructs

**Fig. 3:**
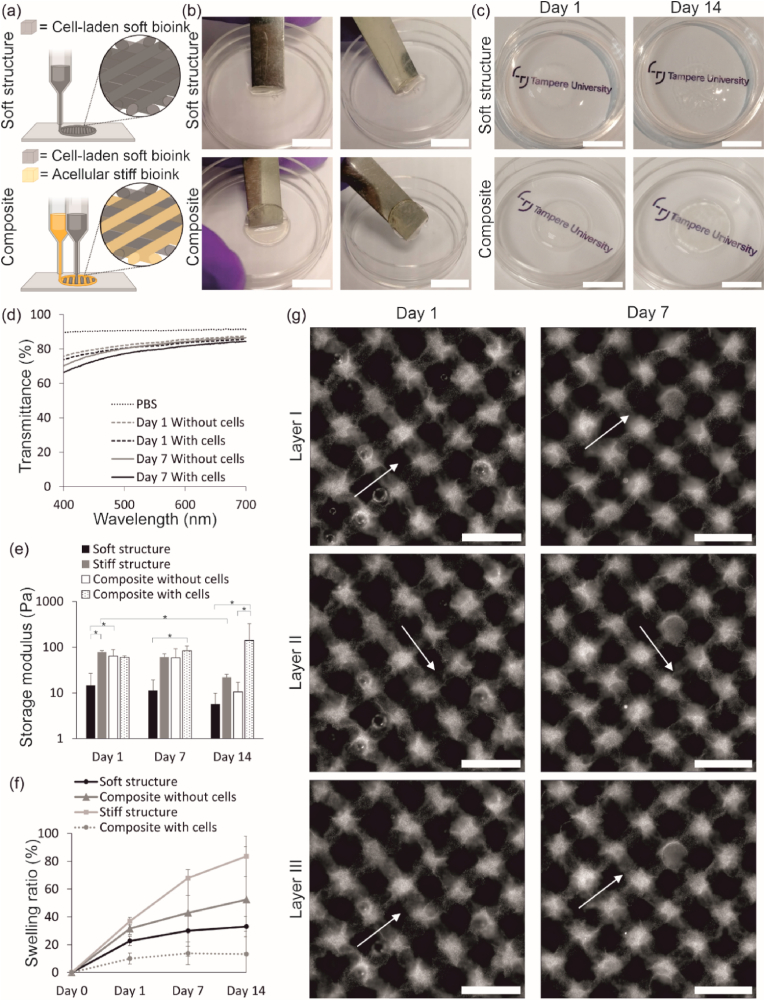
Cell growth increased the mechanical properties of the bioprinted structures. (a) Schematic illustrations of both 3D bioprinted structure types. (b) The acellular composite demonstrated excellent handling ability on day 7 post-printing compared to the soft structure. (c) Cell-laden composite did not shrink or lose visual transparency during culture. (b–c) Scalebars 10 mm. (d) Transmittance was slightly decreased during culture. (e) Cells had a significant effect on the mechanical properties by increasing the storage modulus during culture (*p ≤ 0.05). (f) Cells decreased the swelling of the bioprinted structure. (g) The soft filament of the composite visualized with fluorescent particles was stable during culture. Scalebars 1 mm.

The difference in the important handling properties between a soft-only uni-material structure and a soft + stiff composite (Fig. 3(a)) was analyzed by printing acellular structures and handling them with a spatula on day 7 after printing (Fig. 3(b)). The soft-only structure convoluted when trying to lift it with a spatula, and therefore, could not be handled. The soft + stiff composite was stable, did not convolute and was easily lifted with a spatula. The transparency of cell-laden soft-only uni-material structure and soft + stiff composite was investigated visually, and the transparency of acellular and cell-laden soft + stiff composite with transmittance measurements. There was no difference in visual transparency between the structures when inspected visually 1 day after printing and the text remained visible below the structure during culture (Fig. 3(c)). However, the proliferating cells caused the structures to become cloudier during 14 days of culture. In addition, the soft-only uni-material structure shrunk significantly during culture (Fig. 3(c) top row), which was not observed in the composite. The transmittance of cell-free composites was 76–87 % on day 1 and 70–87 % on day 7, and the transmittance of cell-laden composites 74–86 % on day 1 and 67–84 % on day 7 (Fig. 3(g)). The transmittance of PBS was measured at 89–91 %. The difference in transmittance between structures with or without cells was only maximum of 2 % on day 1 and maximum of 3 % on day 7. A slight decrease in transmittance from day 1 to day 7 occurred regardless of cells, and the decrease was maximum of 5 % without cells and 7 % with cells. Even though the transmittance decreased, it remained still above 66 %.

With follow-up culture up to 14 days, it was observed that the cells had a significant effect on the mechanical properties (p < 0.05) (Fig. 3(e)) and swelling behavior (Fig. 3(f)). The storage modulus demonstrating the mechanical strength of the structures decreased in the case of acellular soft-only and stiff-only uni-material structures, as well as acellular composite without cells. The storage moduli on day 1, 7 and 14 were 14.63 ± 12.13 Pa, 11.28 ± 7.80 Pa and 5.71 ± 4.21 Pa for the acellular soft-only structure, respectively, and 78.59 ± 5.55 Pa, 60.93 ± 10.34 Pa and 21.96 ± 3.74 Pa for the acellular stiff-only structure, respectively. The storage modulus of the acellular soft + stiff composite remained between the moduli of soft-only and stiff-only structures on each timepoint (64.28 ± 23.78 Pa on day 1, 58.55 ± 34.18 Pa on day 7, and 10.5+±6.53 Pa on day 14). However, the storage modulus of the cell-laden soft + stiff composite increased during inspected timepoints (59.78 ± 4.98 Pa on day 1, 83.62 ± 22.79 Pa on day 7, and 141.41 ± 184.36 Pa on day 14). On day 1, the storage modulus of cell-laden composite was similar to the acellular composite. However, on day 14, the storage modulus was 14 times higher in cell-laden composites compare to the acellular composites (p < 0.05). The swelling behavior supported the differences seen in the mechanical properties. Interestingly, the soft-only structure showed lower swelling than the stiff-only structure or the acellular composite, although lower crosslinking density usually results in higher swelling [44]. However, the data could indicate faster material degradation due to lower crosslinking density resulting in lower amount of material, and thus, decreased swelling. The results of the mechanical properties of the structures support this data because the storage modulus of the soft-only structure is significantly lower in each timepoint compared the moduli of other structure types (p ≤ 0.05). The cell-laden composite showed decreased swelling compared to the other explored structures. This data indicates that the cellular component in the bioink has a significant effect on the mechanical properties and swelling behavior of the bioprinted structures. The filament structure within the composite was evaluated by printing fluorescent particles in the soft bioink (Fig. 3(d)). The soft bioink was chosen for the visualization because its organization is more likely to be altered by the stiffer filament, which would result in nonuniform filaments. However, the soft filament was observed to be continuous within the layers, and the structure did not change during the 7 days *in vitro* indicating that the composites maintain their printed form. Importantly, the filament structure mimics the organization of the collagen fibrils in the native corneal stroma where the fibrils are arranged in layers perpendicular to each other [1].

### 3.3 The complex organization of corneal stroma can be mimicked with the multi-material bioprinting strategy

**Fig. 4:**
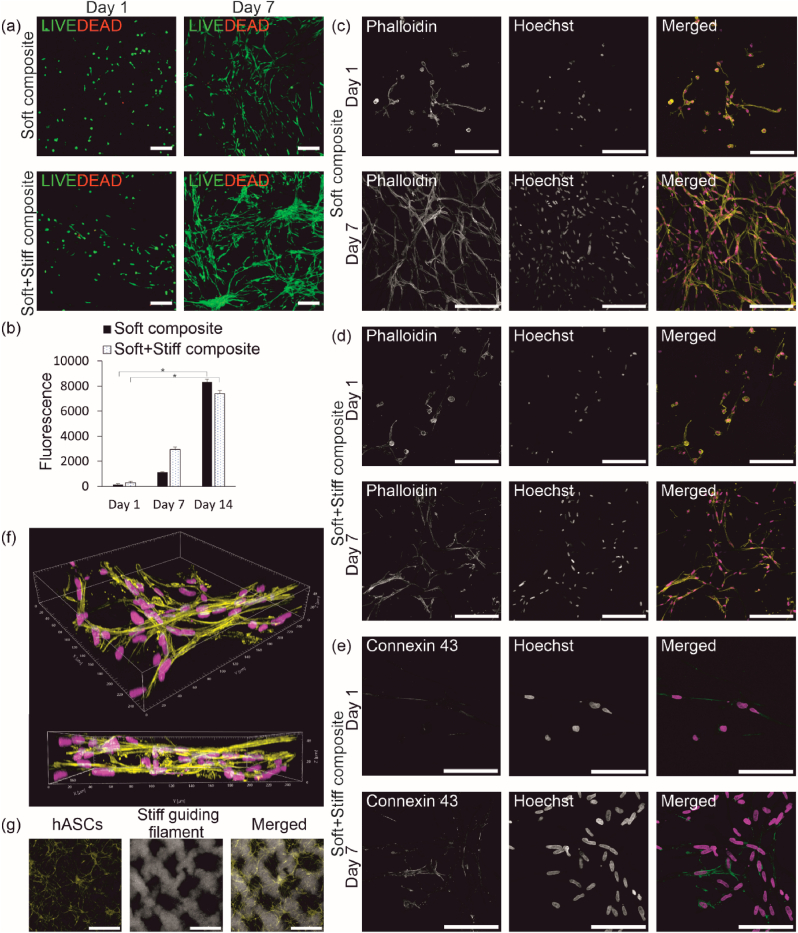
Cytocompatibility and cell growth within the bioprinted composite. (a) Viability of the bioprinted hASCs on day 1 and 7 after printing (green = live cells, red = dead cells). (b) Proliferation of the bioprinted hASCs on day 1 and 7 (*p ≤ 0.05). Cell growth and orientation within (c) the soft composite and (d) the soft + stiff composite on day 1 and 7. Cell morphology illustrated with phalloidin (yellow). (e) Cell-cell interactions within the soft + stiff composite on day 1 and 7 visualized with gap junction protein connexin 43 (green). (f) 3D illustration of the orientation and growth of hASCs within the soft + stiff composite on day 7 with phalloidin (yellow). (g) A macroscopic illustration of the cell growth and orientation with phalloidin (yellow) and fluorescent particles (white) on day 7. Nuclei visualized with Hoechst (magenta, (*c*–f)). Illustration with confocal maximum intensity projection images ((a), (*c*–e), (g)). Scalebars 200 μm ((a), (*c*–d)), 100 μm (e) and 1 mm (g).

The ability of the multi-material 3D bioprinting strategy to guide cellular growth in the bioprinted structures was explored by printing cell-laden composites using either stiff or soft bioink as the acellular bioink (Fig. 1(c)). The cells were printed in the soft bioink with both structure types. Cytocompatibility of the multi-material bioprinting strategy was studied with LIVE/DEAD staining on day 1 and 7 post-printing. High cell viability (>98 %) with hardly any dead cells was observed in both printed structure types (Fig. 4(a)). The cell proliferation within the two composites was further studied with PrestoBlue™ analysis on day 1, 7 and 14 post-printing. The proliferation increased significantly during 14 days of culture (p ≤ 0.05), and there was no significant difference between the different printed composites in any timepoint (Fig. 4(b)). This data indicates excellent cytocompatibility of the bioinks with hASCs. Importantly, the multi-material bioprinting did not hamper the cell viability during the printing process.

The cell growth and tissue formation were further studied in the printed composites with IF staining. Phalloidin staining did not show visible differences in cell morphology or orientation on day 1 post-printing (Fig. 4(c)). In addition, the cells were in the cell-laden soft filament with both structure types. The cell morphology was already elongated to some degree on day 1. Completely elongated cells were detected on day 7 with no rounded cells visible (Fig. 4(d)). However, cells were no longer within the original filament organization with the soft-only composite and proliferated within the whole structure. With the stiff filament guiding the cell growth, the structural organization remained during 7 days of culture. However, it was observed that after 7 days, the organization was gradually lost due to high cell proliferation in the printed structures (Fig. S1). Tissue formation within the soft + stiff composite was evaluated with IF staining of gap junction protein connexin 43 (Fig. 4(e)). Positive staining against connexin 43 was observed on day 1 and 7 after printing, with increased expression during culture. This indicates an increase in cell-cell interactions within the printed structure. In addition, the cellular organization and network formation in layer-like fashion was observed in the 3D illustration of a confocal image (Fig. 4(f)). The effect of the multi-material 3D bioprinting strategy on cell growth was further illustrated by adding fluorescent particles to the stiff filament and staining the cells with phalloidin. The cells grew along the stiff filament and formed network-like structures (Fig. 4(g)).

### 3.4 Integration of the 3D bioprinted composite to the host tissue

**Fig. 5:**
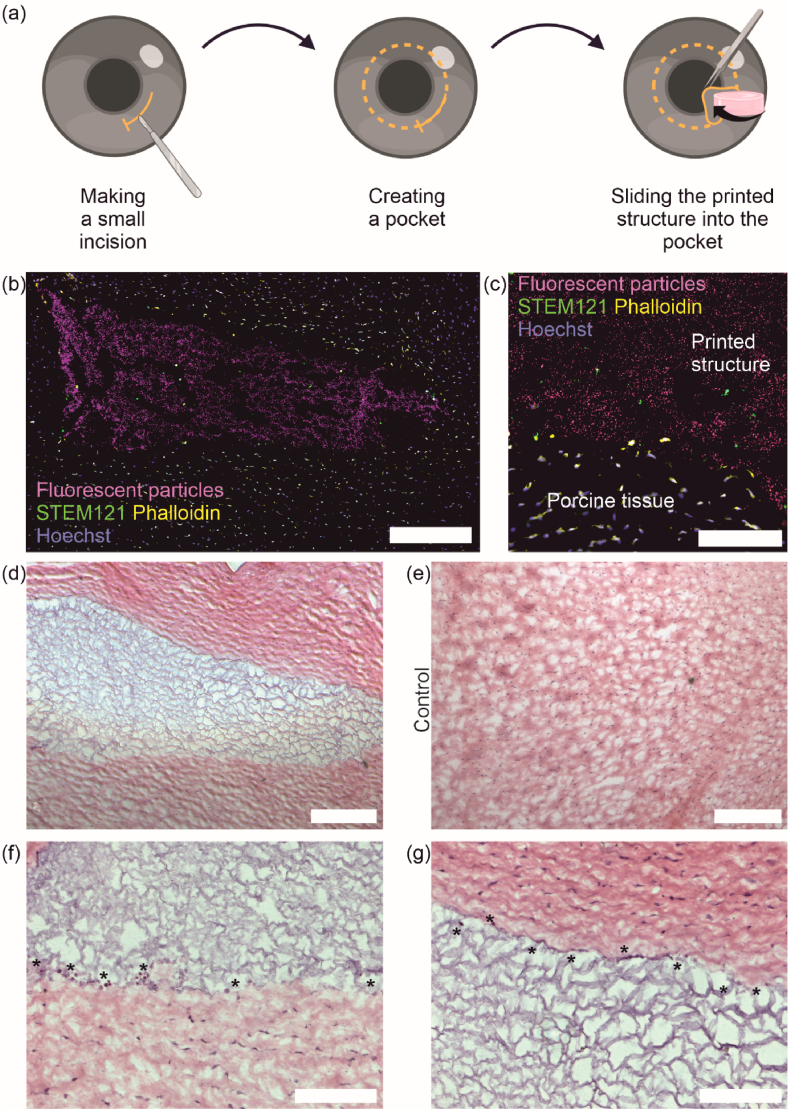
3D bioprinted composites in porcine *ex vivo* culture. (a) Schematic illustration of transplanting a bioprinted composite into a corneal stromal pocket. (b–c) Day 14 integration of the bioprinted composite to the host tissue visualized from cryosections with fluorescent particles (magenta) and IF staining of human cell specific marker STEM121 (green), phalloidin (yellow) and Hoechst (blue). (d) Day 14 H&E staining of the bioprinted structure in corneal stromal pocket. (e) H&E staining of control porcine cornea on day 14. (f–g) Higher magnification images of day 14 H&E staining visualizing cells (*) interacting between the bioprinted composite and the host tissue. Scalebars 1 mm (b), 200 μm (*c*–e) and 100 μm (f–g).

After demonstrating the cellular growth within the 3D bioprinted composite, the integration of the 3D structure to *ex vivo* porcine cornea was investigated to avoid unnecessary animal studies. The bioprinted cell-laden soft + stiff composites were transplanted after 4 h post-printing into stromal pockets as shown in Fig. 5(a). The integration was evaluated after 14 days in culture with IF and H&E staining from cryosections (Fig. 5(b–g)). Importantly, the bioprinted composites were easily transplanted without additional support, sutures, or tissue glue. In IF staining, human stem cell marker STEM121 was used to visualize the human cells from the bioprinted composite (Fig. 5(b and c), green). Fluorescent particles mixed in the bioink were used to detect the whole bioprinted composite in the *ex vivo* model (Fig. 5(b and c), magenta). Fluorescent particles and cells negative for STEM121 were seen attached to the bioprinted composite (Fig. 5(c), arrows indicating STEM121-negative cells). This could indicate that the bioprinted composite is attached to the surrounding porcine *ex vivo* tissue and the STEM121-negative porcine cells are migrating towards the bioprinted composite. Moreover, H&E staining of the porcine cornea *ex vivo* cryosection with the bioprinted composite (Fig. 5(d)) shows tight attachment between the composite and host tissue. The composite (purple) is in contact with the surrounding tissue (red) all around the structure without ruptures. In addition, based on the H&D staining, the architecture of the control porcine *ex vivo* cornea (Fig. 5(e)) is similar to the one with the bioprinted composite (Fig. 5(d)). In higher magnification images of H&E staining of the bioprinted composite in the corneal stromal pocket (Fig. (f–g)), tight attachment of the bioprinted structure to the porcine cornea *ex vivo* tissue can be seen with cells (*) are located in between the bioprinted structure and the host tissue and across the sample edge.

## 4 Discussion

Multi-material 3D bioprinting revolutionizes medical additive manufacturing by shifting the paradigm of bioprinting from simple uni-material structures to complex, native-like tissue constructs. The heterogenous composition and organization of the corneal stroma is fundamental for the normal structure and function of the human cornea [1] and its disruptions can lead to corneal blindness. Donor corneas are scarce, and thus, artificial corneas are in desperate need. Here, we developed a novel multi-material 3D bioprinting strategy for bioprinting corneal stroma structures. To mimic the native microstructure of the corneal stroma, cell-laden and acellular bioinks with varying stiffness were bioprinted in alternating filaments and perpendicular layers to create composites. This innovative strategy builds upon prior work where multi-material bioprinting of hydrogel bioinks has been mostly explored to provide spatial placement of different cell types without guidance for their growth in microscale upon maturation [16,22]. Therefore, our approach advances 3D bioprinting of cornea and other complex human tissues. In addition to achieving the native-like microstructure, the effects of the multi-material bioprinting strategy on cellular growth after printing as well as the handling and mechanical strength of the structures were investigated. This was the first time the multi-material extrusion-based bioprinting of bioinks with different mechanical properties was explored in cornea TE.

In the previous research done by others, the 3D bioprinting of corneal stroma mimicking structures have been explored only with the uni-material approach applied to extrusion-based [5,6], inkjet-based [7] or stereolithography-based bioprinting [9,10]. However, achieving the complex and heterogenous nature of human tissues requires bioprinting multiple bioinks with different mechanical and biological properties [14]. In our previous study, we explored the multi-material approach in laser-assisted bioprinting by printing alternating layers of acellular and cell-containing bioinks [8]. Even though this approach led to corneal stroma mimicking structures in cross sections, the cellular organization within layers was not fully achieved. In addition, the mechanical properties of the printed structures were not sufficient to withstand handling as such and additional supportive membrane was required for handling. Building on this research, here we focused on achieving the heterogenous microstructure and layered organization, providing a disruptive solution to manufacture corneal stromal structures that withstand mechanical handling.

One of the enduring challenges in bioprinting is developing bioinks that fulfill the physicochemical requirements for their printing application, as well as the biological requirements associated with the embedded cells [45]. Prior research generally confirms that high mechanical stress, such as shear stress during bioprinting process, increase cell death [46,47]. Shear stress is affected by the printing nozzle diameter, printing pressure and viscosity of the bioink [47]. The resolution in extrusion-based bioprinting is typically limited in the range of hundred micrometers to millimeters [45] resulting in limited biomimicry. To challenge the resolution limit and to mimic the native-like collagen fibril organization of corneal stroma, we used a 100 μm printing nozzle. Due to the small printing nozzle, we designed a low viscosity bioink for bioprinting hASCs with low printing pressure. This bioink demonstrated shear-thinning properties also with cells which is important in extrusion-based bioprinting to reduce shear stress and prevent cell death [45]. The viscosity of the cell-laden soft bioink was similar to the viscosity of the acellular soft ink, the difference in the average peak viscosities being only 7 Pa s. This indicates that the cells do not affect the viscosity or shear-thinning of the soft bioink, at least with the relatively low cell density of 0.9 million cells ml^−1^ that was used in this study. Consequently, high viability of hASCs was achieved after printing which demonstrates the ability of the bioink to protect the delicate stem cells during printing. In addition to protecting stem cells from shear stress during bioprinting, suitable mechanical properties after printing are also vital. It is well known that the mechanical properties of the environment affect the cellular functions [48] and stem cell behavior [49]. Importantly, bioinks with high mechanical strength do not necessarily allow stem cell migration or growth resulting in poor tissue formation in printed structures [46]. Therefore, bioprinting human stem cells requires softer bioinks that allow cellular growth and interaction since cell proliferation, cellular interactions and tissue formation are essential to manufacture functional bioprinted tissues. Previously in corneal bioprinting, the research focus has been on evaluating the cellular viability rather than demonstrating cellular interactions or tissue formation after printing [[5], [6], [7],[9], [10], [11]]. Here, by bioprinting hASCs in the soft bioink with low crosslinking density, we achieved high cell proliferation and expression of gap junction protein connexin 43, indicating that the soft bioink supports tissue formation. Moreover, the cell morphology and connexin 43 expression in the soft composite on day 7 was similar as was previously seen in soft-only uni-material structures (Fig. S2). This is in line with our previous results with a bioink with slightly higher crosslinking degree [28]. Consequently, the soft bioink was favorable for stem cell bioprinting and shows great promise for additive manufacturing of stem cells.

Despite great cytocompatibility and tissue formation of hASCs in the soft bioink, its mechanical properties as such were not sufficient for fulfilling the physicochemical requirements for cornea TE. The structures bioprinted only with the soft bioink suffered from inadequate mechanical properties for handling and shrinkage during culture due to cell proliferation. Employing multi-material bioprinting strategy to manufacture composites with the cell-laden soft bioink and the stiff acellular bioink, the handling of the structures improved significantly which is vital for the transplantation of artificial corneas. Moreover, no shrinking of the cell-laden composites was observed during *in vitro* cultures. Importantly, the multi-material approach did not hinder the cell viability or proliferation after printing. Consequently, the multi-material bioprinting strategy allowed us to combine the distinct mechanical properties of bioinks without compromising the cellular growth and tissue formation and furthermore, opened the opportunity to guide cellular growth in 3D bioprinted structures and achieve the heterogenous design. Uni-material bioprinting approach cannot achieve and maintain the highly organized arrangement of cells and ECM which is crucial for fabricating transparent native corneal stroma mimicking structures. This was also detected here as there was no cellular organization without the stiff acellular bioink. Therefore, alternating filaments of cell-laden soft bioink with acellular stiff bioink were printed to demonstrate the cellular organization after printing, and the printed filaments were perpendicular in the adjacent layers to mimic the lamellar structure of the native cornea stroma. Herein, it was shown that the cellular growth was more organized when stiff bioink was used in the guiding acellular filaments. However, the organization decreased after one week of culture. At this timepoint, the composites were stable with intact filaments indicating that the decrease in the cellular organization is due to high proliferation of hASCs. In native cornea, hCSKs are quiescent with extremely low proliferation [50]. In addition, stem cell differentiation is known to reduce their proliferation capacity [51], and we have observed the decrease in the proliferation capacity in our previous research when differentiating hASCs towards hCSKs [28]. Thus, bioprinting of hCSKs or hASCs differentiated towards hCSKs with lower proliferation could improve the maintenance of organized cellular structures in long-term cultures in future studies.

Bioink transparency is a prerequisite in corneal applications and advantageous for other TE applications to allow constant monitoring and imaging of the 3D bioprinted structures during tissue maturation. In previous research on heterogenous microstructure of corneal stroma, combination of biofabrication technologies have been explored. Fernández-Pérez et al. [52] electrospun PCL and porcine cornea -derived ECM to scaffolds with varying fiber organization and Gao et al. [18] used MEW technology and PCL for corneal stroma TE. These studies showed great advances in cell organization of corneal stromal keratocytes. However, the use of abundant non-transparent PCL fibers results in scattering of light and loss of transparency. In addition, manual cell seeding is required in both biofabrication technologies, causing limitations in the spatial distribution of the cells and limiting the scalability and automatization of these processes. To overcome these issues, we combined the multi-material 3D bioprinting strategy with the bioinks with proper transparency. Both bioinks demonstrated transmittance above 75 % throughout the wavelength range of visible light, which is considered excellent in corneal transparency classification [53]. In addition, the transmittance value for human cornea is reported to range from 75 % to 90 % in the visible light wavelength range [54]. Despite the bioink transparency being sufficient for corneal applications, high hASC proliferation led to cloudy appearance of the bioprinted structures after 14 days of culture. Similar phenomenon with 3D bioprinted hASCs has been reported previously with laser-assisted 3D bioprinting [8]. This phenomenon was detected in the transmittance measurements of bioprinted structures with and without cells as the transmittance decreased slightly. However, the transmittance of cell-laden composite day 7 was still excellent or above reasonable (67–84 %) in corneal transparency classification [53], indicating that the cell proliferation did not significantly hinder the transparency. Furthermore, bioprinting hCSKs or hASCs differentiated towards hCSKs with lower proliferation and maintaining the cellular organization in long-term cultures could also enhance the transparency during culture. Moreover, corneal transparency is decreased if swelling occurs [54]. Yazdanpanah et al. [55] pointed out that biomaterials used in corneal TE need to have low swelling. Here, the swelling of the bioprinted cell-laden composite was detected to be lower compared to the acellular structure types. This can be due to the high cell proliferation and tissue formation occurring after printing which leads to decreased amount of porosity and less space to swell. However, the long-term transparency and degradation of bioprinted structures should be investigated in corneal *in vivo* model in future studies.

In addition to transparency, the mechanical strength of the corneal stroma is one of its key properties [1]. The average storage modulus of native porcine corneal stroma has been reported to range from 2 to 8 kPa when studying the effect of increasing compressive modulus on the storage modulus [56]. Even though the developed multi-material bioprinting strategy demonstrated significant improvement in handling, and thus potential for transplantation, the storage moduli of acellular and cell-laden composites on day 1 post-printing were 0.064 ± 0.024 kPa and 0.069 ± 0.005 kPa, respectively. Therefore, the values are significantly lower than in the native corneal stroma. Moreover, the storage moduli of all the acellular structure types decreased upon culture post-printing. However, interestingly, the storage modulus of cell-laden composite structure increased up to 0.141 ± 0.184 kPa after 14 days of culture. Even though the value is still lower than the values reported for the native cornea stroma, the data reported here demonstrates that when the cells can proliferate, alter the bioprinted environment and interact, the mechanical strength increases upon culture. The increase in the mechanical strength of cell-loaded 3D bioprinted structures during culture has also been demonstrated previously with primary hCSKs and gelatin methacrylate bioink [6]. Therefore, the bioink itself does not necessarily need to match the mechanical requirements of the native tissue. In addition, it has been previously suggested that it may not be necessary for bioengineered tissue to match all the mechanical properties of the native cornea to be therapeutically effective [57]. However, this suggestion should be validated *in* *vivo* setting in future studies with cell-laden composites.

Due to the employment of multiple materials in the composites, material interactions at the interfaces must be considered. Strong interfacial adhesion improves the toughness and fatigue-resistance of bioprinted constructs [58]. The sufficient handling of the composites indicated successful adhesion of alternating filaments, and this was further confirmed by a gel block fusion test and compression test. This finding of strong interfacial adhesion was supported by the mechanical characterization, where the storage modulus of the acellular composite was in the similar range as with the stiff-only uni-material structures. Thus, the multi-material 3D bioprinting strategy is a highly potential solution for fabricating mechanically robust, heterogeneously designed, cell-laden solid 3D structures. The strategy also supports tissue formation and guides cellular growth, and therefore it is a crucial technological advancement for creating artificial corneas with native-like microstructure.

After demonstrating the advantages of the multi-material 3D bioprinting strategy compared to uni-material approaches in corneal TE, we explored the integration of the bioprinted composite to host tissue in porcine *ex vivo* cornea organ culture model. Integration of the artificial cornea to the host tissue is crucial to prevent graft failure. The advantages of *ex vivo* tissue models have been demonstrated in several different tissues, such as cornea [59], skin [60], cartilage [61] and bone [62]. Available *ex vivo* models are economical and ethical approaches to study the interaction between the host tissue and the transplanted material. Importantly, unnecessary animal studies can be avoided with *ex vivo* models. Here, the transplantation of the composites was successfully done in stromal pockets, which is a generally used surgical technique to study biocompatibility of the bioengineered corneal implants *in vivo* [[63], [64], [65], [66], [67]]. After transplantation, the composite remained in place during *ex vivo* organ culture. Moreover, porcine cells from the native tissue (negative for human stem cell marker STEM121) were seen attached to the bioprinted composite already after 14 days. This indicates migration of the porcine cells towards the bioprinted composite. Migration of host cells and strong stromal adhesion of bioengineered corneas *in ex vivo* models have been previously reported to be indicative of tissue biocompatibility [57]. In addition, the architecture of the 3D bioprinted composites resembled the architecture of the native cornea stroma in H&E staining. The composites were tightly attached to the host porcine stroma. These results indicate good integration of the bioprinted composite to the host tissue. However, long-term *in vivo* performance and integration assessment is needed in future to fully evaluate the suitability of these multi-material 3D bioprinted composites for cornea TE.

## 5 Conclusions

Multi-material 3D bioprinting will revolutionize the field of translational additive manufacturing since uni-material bioprinting approaches cannot mimic the heterogenous nature of native human tissues. This study advances the research in additive manufacturing of human tissue constructs with heterogenous design specifically in the field of corneal TE. Here, a novel multi-material 3D bioprinting strategy was developed using HA-based bioinks with varying stiffnesses. The developed multi-material bioprinting strategy was applied in 3D bioprinting human corneal stroma. The combination of soft and stiff bioink resulted in 3D bioprinted structures with good physicochemical and biological properties for corneal TE applications. Bioprinting a cell-laden soft bioink together with an acellular stiff bioink into alternating filaments and perpendicular layers allows mimicking the organization of the heterogenous microstructure in the corneal stroma. In addition, the soft bioink promoted cellular growth and tissue formation of human stem cells in the multi-material 3D bioprinted composites, whereas stiff bioink provided mechanical support as well as guidance of cellular organization upon culture. This was the first study where multi-material 3D bioprinting strategy was explored for 3D bioprinting of the human corneal stroma. Therefore, it holds great potential as a biofabrication solution for manufacturing organized, heterogenous microstructures of native tissues *in vitro*.

## Funding

This work was supported by grants from the 10.13039/501100004012Jane and Aatos Erkko Foundation (HS and 10.13039/100011405AM, 2020); 10.13039/501100002341Academy of Finland (324082 10.13039/100011405AM, 336666 SM, 326588 SM, 312413 SM, 337607 SM); 10.13039/501100003125Finnish Cultural Foundation (PP); and 10.13039/501100013510Eye and Tissue Bank Foundation (PP).

## CRediT authorship contribution statement

**Paula Puistola:** Conceptualization, Investigation, Methodology, Visualization, Writing - original draft, Writing - review & editing. **Susanna Miettinen:** Resources, Writing - review & editing. **Heli Skottman:** Funding acquisition, Project administration, Resources, Supervision, Writing - review & editing. **Anni Mörö:** Conceptualization, Funding acquisition, Methodology, Project administration, Supervision, Writing - review & editing.

## Declaration of competing interest

The authors declare the following financial interests/personal relationships which may be considered as potential competing interests:Heli Skottman reports financial support was provided by Jane and Aatos Erkko Foundation. Anni Mörö reports financial support was provided by 10.13039/501100002341Academy of Finland. Susanna Miettinen reports financial support was provided by 10.13039/501100002341Academy of Finland. Paula Puistola reports financial support was provided by 10.13039/501100003125Finnish Cultural Foundation. Paula Puistola reports financial support was provided by 10.13039/501100013510Eye and Tissue Bank Foundation. Anni Mörö reports a relationship with StemSight Oy that includes: equity or stocks. Heli Skottman reports a relationship with StemSight Oy that includes: equity or stocks. Anni Mörö has patent #PCT/FI2022/050,403 pending to Assignee. Based on the Act on the Right in Inventions in Finland, all authors employed by Tampere University have given all rights to the University and thus have declared no competing interests. Anni Mörö and Heli Skottman are co-founders and shareholders in StemSight Ltd without any connection to the technology and results reported in this manuscript. The other authors declare no conflicts of interests.

## Data Availability

Data will be made available on request.
