# Supplementary material for: Novel strategy for multi-material 3D bioprinting of human stem cell based corneal stroma with heterogenous design
Source: Mater Today Bio. 2023 Dec 22;24:100924. doi: 10.1016/j.mtbio.2023.100924 (PMC10788621; doi:10.1016/j.mtbio.2023.100924)

**Appendix A. Supplementary data**

Supplementary immunofluorescence data in Fig. S1 and Fig. S2.

Fig. S1. Cell growth and orientation within the soft + stiff composite on day 14. (a) Cell morphology illustrated with phalloidin (yellow). Scalebars 200 µm. (b) Cell-cell interactions visualized with gap junction protein connexin 43 (green). Scalebars 100 µm. Nuclei visualized with Hoechst (magenta, (a-b)).


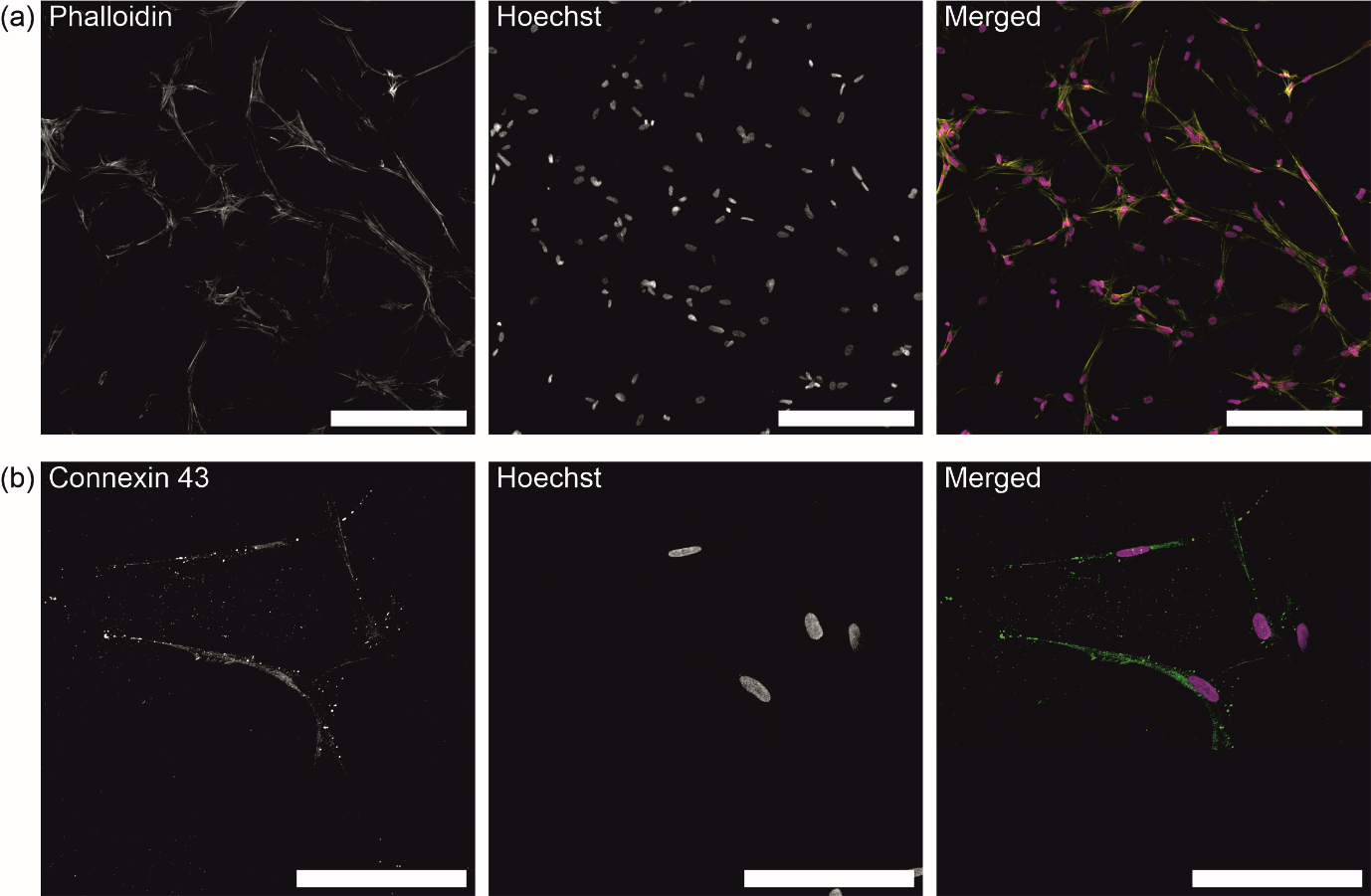


Fig. S2. Cellular interactions in the soft-only uni-material structure on day 7. Cell-cell interactions visualized with gap junction protein connexin 43 (green). Cell morphology illustrated with phalloidin (yellow). Nuclei visualized with Hoechst (magenta). Scalebars 200 µm.


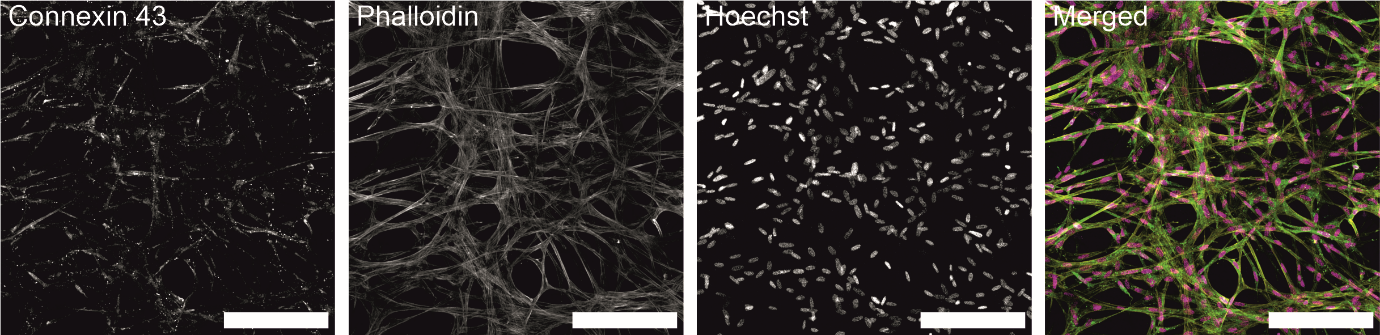

Supplement: Multimedia component 1 [file mmc1.docx]
